# Supplementary material for: Associations of Bone Mineral Density with RANKL and Osteoprotegerin in Arab Postmenopausal Women: A Cross-Sectional Study
Source: Medicina (Kaunas). 2022 Jul 22;58(8):976. doi: 10.3390/medicina58080976 (PMC9330386; doi:10.3390/medicina58080976)
Supplement: Supplementary file 1 [file medicina-58-00976-s001.zip › medicina-1777581-supplementary.pdf]

Table S1. Clinical Characteristics of Participants according to Tertile 1 (Subjects ≤ 54.0 years)

Parameters	Age ≤ 54.0 years			
	Normal	Osteopenia	Osteoporosis	P-value
N	78	98	51	
Age (year)	51.0 ± 3.3	51.2 ± 2.5	50.0 ± 4.7	0.14
Age of Menarche	13.0 ± 1.4	13.2 ± 1.6	13.7 ± 1.7	0.03
Age at First Pregnancy	19.5 ± 3.9	18.6 ± 3.4	20.0 ± 3.7	0.08
Years since Menopause	7.8 ± 12.6	7.9 ± 11.0	10.3 ± 12.4	0.42
BMI (kg/m^2)	33.7 ± 5.4	33.4 ± 5.0	29.5 ± 6.2^{AB}	<0.001
WHR	0.91 ± 0.10	0.92 ± 0.09	0.89 ± 0.09	0.28
BMD (Spine)	1.2 ± 0.1	1.0 ± 0.1^{A}	0.8 ± 0.1^{AB}	<0.001
BMD (Femoral neck left)	1.1 ± 0.1	0.9 ± 0.1^{A}	0.8 ± 0.1^{AB}	<0.001
T-Score (Spine)	0.1 ± 0.9	-1.6 ± 0.6^{A}	-2.9 ± 0.4^{AB}	<0.001
RANKL (pg/mL)	44.0 (25.5 – 77.9)	34.8 (19.6 – 64.4)	34.3 (22.1 – 44.9)	0.23
OPG (pg/mL)	678.0 (546.8 – 907.8)	692.9 (498.5 – 1016.1)	773.6 (602.4 – 1041.8)	0.79
RANKL/OPG	0.1 (0.0 – 0.1)	0.0 (0.0 – 0.1)	0.0 (0.0 – 0.1)	0.43
Osteocalcin (ng/mL)	9.9 (3.3 – 15.1)	8.5 (2.7 – 13.2)	6.8 (2.1 – 13.0)	0.61
NTx (nmol)	50.4 (41.0 – 68.2)	53.4 (42.4 – 101.0)	59.1 (43.0 – 95.3)	0.67
Testosterone (ng/mL)	0.6 (0.4 – 1.5)	0.4 (0.3 – 0.7)	0.9 (0.3 – 1.1)	0.34
Estradiol (pg/mL)	64.6 (41.0 – 121.7)	63.7 (34.8 – 162.3)	77.3 (33.3 – 185.5)	0.99

Note: Data Presented as Mean ± SD and Median (25st -75th) percentiles for Gaussian and Non-Gaussian variables. The superscripts A and B means significant difference compared to normal and osteopenia groups respectively. P-value was considered significant at 0.05 and 0.01 levels.

Table S2. Clinical Characteristics of Participants according to Tertile 2 (Subjects aged 55-60)

Parameters	Tertile 2: Age 55-60			
	Normal	Osteopenia	Osteoporosis	P-value
N	58	107	65	
Age (year)	57.4 ± 2.0	57.2 ± 2.1	57.8 ± 2.0	0.12
Age of Menarche	13.1 ± 1.2	13.4 ± 1.5	13.5 ± 1.6	0.38
Age at First Pregnancy	19.3 ± 3.8	18.6 ± 3.4	19.7 ± 4.4	0.22
Years since Menopause	11.0 ± 13.7	10.7 ± 11.4	14.0 ± 14.1	0.23
BMI (kg/m^2)	34.8 ± 5.9	33.3 ± 5.5	32.3 ± 5.9	0.07
WHR	0.93 ± 0.12	0.91 ± 0.09	0.93 ± 0.10	0.43
BMD (Spine)	1.2 ± 0.1	1.0 ± 0.1 ^{A}	0.8 ± 0.1 ^{AB}	<0.001
BMD (Femoral neck left)	1.0 ± 0.1	0.9 ± 0.1 ^{A}	0.8 ± 0.1 ^{AB}	<0.001
T-Score (Spine)	-0.2 ± 0.7	-1.7 ± 0.5 ^{A}	-3.0 ± 0.4 ^{AB}	<0.001
RANKL (pg/mL)	44.1 (21.2 – 92.9)	30.9 (18.2 – 55.4)	30.6 (22.9 – 62.1)	0.52
OPG (pg/mL)	888.0 (605.6 – 1028.8)	826.1 (599.0 – 986.8)	782.2 (641.2 – 1022.8)	0.94
RANKL/OPG	0.0 (0.0 – 0.1)	0.0 (0.0 – 0.1)	0.0 (0.0 – 0.1)	0.48
Osteocalcin (ng/mL)	11.9 (3.3 – 15.3)	7.7 (2.7 – 14.7)	9.6 (4.8 – 11.9)	0.57
NTx (nmol)	58.8 (49.9 – 74.4)	49.8 (34.9 – 66.0)	55.4 (31.9 – 65.1)	0.31
Testosterone (ng/mL)	0.8 (0.7 – 1.0)	0.6 (0.4 – 1.0)	0.7 (0.5 – 1.0)	0.31
Estradiol (pg/mL)	76.1 (42.2 – 200.4)	65.5 (33.0 – 178.8)	71.4 (32.4 – 270.1)	0.84

Note: Data Presented as Mean ± SD and Median (25st -75th) percentiles for Gaussian and Non-Gaussian variables. The superscripts A and B means significant difference compared to normal and osteopenia groups respectively. P-value was considered significant at 0.05 and 0.01 levels.

Table S3. Clinical Characteristics of Participants according to Tertile 3 (Subjects aged ≥ 61)

Parameters	Tertile 3 Age ≥ 61			
	Normal	Osteopenia	Osteoporosis	P-value
N	30	77	53	
Age (year)	66.3 ± 3.8	66.7 ± 5.2	67.6 ± 5.3	0.47
Age of Menarche	13.2 ± 1.5	13.5 ± 1.6	13.1 ± 1.7	0.42
Age at First Pregnancy	18.7 ± 3.7	18.4 ± 3.1	18.5 ± 3.8	0.95
Years since Menopause	15.7 ± 12.1	14.7 ± 8.6	17.7 ± 8.4	0.19
BMI (kg/m^2)	33.1 ± 6.2	33.5 ± 6.2	28.5 ± 6.0^{AB}	<0.001
WHR	0.93 ± 0.10	0.94 ± 0.08	0.93 ± 0.09	0.64
BMD (Spine)	1.2 ± 0.1	1.0 ± 0.1 ^{A}	0.8 ± 0.1 ^{AB}	<0.001
BMD (Femoral neck left)	1.0 ± 0.1	0.9 ± 0.1 ^{A}	0.7 ± 0.1 ^{AB}	<0.001
T-Score (Spine)	-0.1 ± 0.5	-1.8 ± 0.5 ^{A}	-3.1 ± 0.9 ^{AB}	<0.001
RANKL (pg/mL)	46.3 (26.7 - 70.5)	30.6 (20.8 - 44.7)	37.4 (22.4 - 71.0)	0.18
OPG (pg/mL)	842.8 (610.6 - 1430.7)	909.9 (792.8 - 1190.3)	1036.1 (961.3 - 1502)	0.32
RANKL/OPG	0.0 (0.0 - 0.0)	0.0 (0.0 - 0.0)	0.0 (0.0 - 0.0)	0.82
Osteocalcin (ng/mL)	14.7 (11.1 - 18.1)	8.8 (4.5 - 14.5) ^{A}	11.1 (9.4 - 14.0)	0.049
NTx (nmol)	71.0 (58.6 - 116.7)	60.8 (53.8 - 71.5)	61.9 (53.4 - 82.8)	0.69
Testosterone (ng/mL)	1.0 (0.5 - 1.6)	0.7 (0.3 - 0.9)	0.3 (0.1 - 0.6)	0.23
Estradiol (pg/mL)	80.8 (36.6 - 124.9)	48.5 (34.6 - 195.7)	49.9 (35.3 - 74.6)	0.90

Note: Data Presented as Mean ± SD and Median (25st -75th) percentiles for Gaussian and Non-Gaussian variables. The superscripts A and B means significant difference compared to normal and osteopenia groups respectively. P-value was considered significant at 0.05 and 0.01 levels.
